# Histopathologic Evaluation of Corneal Tissue After Adjunctive Rose Bengal Photodynamic Antimicrobial Therapy and Keratoplasty in Advanced Acanthamoeba Keratitis

**DOI:** 10.3390/jcm14176104

**Published:** 2025-08-29

**Authors:** Jordan J. Huang, Juan Carlos Navia, Joshua M. Huang, Matthew Camacho, Charissa H. Tan, Paula A. Sepulveda-Beltran, Sara Mustafa, Heather Durkee, Alejandro Arboleda, Mariela C. Aguilar, Darlene Miller, Jean-Marie Parel, Guillermo Amescua, Sander R. Dubovy, Jaime D. Martinez

**Affiliations:** 1 Department of Ophthalmology, Bascom Palmer Eye Institute, University of Miami Miller School of Medicine, 900 NW 17th St., Miami, FL 33136, USA; jhuan026@uottawa.ca (J.J.H.); jcnavia@miami.edu (J.C.N.); joshua11@ualberta.ca (J.M.H.); mcamach4@uthsc.edu (M.C.); charissa.tan@utah.edu (C.H.T.); sara4mustafa@gmail.com (S.M.); alejandro.arboleda@bcm.edu (A.A.); dmiller@med.miami.edu (D.M.); gamescua@med.miami.edu (G.A.); sdubovy@med.miami.edu (S.R.D.); 2 Ophthalmic Biophysics Center, Department of Ophthalmology, Bascom Palmer Eye Institute, University of Miami Miller School of Medicine, 1638 NW 10th Ave., Miami, FL 33136, USA; hdurkee@med.miami.edu (H.D.); maguilar1@med.miami.edu (M.C.A.); jmparel@med.miami.edu (J.-M.P.); 3 Florida Lions Ocular Pathology Laboratory, Department of Ophthalmology, Bascom Palmer Eye Institute, University of Miami Miller School of Medicine, 900 NW 17th St., Miami, FL 33136, USA; 4 Ocular Microbiology Laboratory, Department of Ophthalmology, Bascom Palmer Eye Institute, University of Miami Miller School of Medicine, 1638 NW 10th Ave., Miami, FL 33136, USA

**Keywords:** rose bengal photodynamic antimicrobial therapy, histopathology, microbiology, penetrating keratoplasty, Acanthamoeba, Acanthamoeba keratitis, Acanthamoeba cyst, therapeutic penetrating keratoplasty, optical penetrating keratoplasty, deep anterior lamellar keratoplasty, corneal infection

## Abstract

**Background/Purpose**: To compare the microbiologic and histopathologic features of Acanthamoeba isolates recovered from patients with Acanthamoeba keratitis (AK) who underwent a therapeutic penetrating keratoplasty (TPK), optical penetrating keratoplasty (OPK), or deep anterior lamellar keratoplasty (DALK) after Rose Bengal Photodynamic Antimicrobial Therapy (RB-PDAT). **Methods**: Surgical specimens were stained with hematoxylin, eosin, and Periodic Acid-Schiff stains as per institutional protocol at the University of Miami, Bascom Palmer Eye Institute. Analysis of Acanthamoeba cyst depth, number of cysts, and average corneal thickness was established by light microscopy. **Results**: Seventeen patients with AK underwent surgical intervention and RB-PDAT. Eight patients underwent a TPK and nine patients underwent an OPK/DALK. In the TPK group, average cyst depth was 42.0 ± 52.5 μm from Descemet’s layer and mean corneal button thickness was 661.7 ± 106.5 μm. Comparatively, in the OPK/DALK group, average cyst depth from Descemet’s layer was 261.7 ± 222.7 μm with a mean corneal button thickness of 474.2 ± 126.6 μm. **Conclusions**: Acanthamoeba cysts were found to penetrate deeper within the cornea amongst patients that underwent an emergent TPK compared to patients that underwent an elective OPK/DALK. This may suggest an association between Acanthamoeba cyst depth and infection severity and provides valuable clinical insights towards understanding factors such as infection recurrence and resistance to treatment.

## 1. Introduction

Acanthamoeba keratitis (AK) is a rare parasitic infection of the eye by a unicellular protozoan of the genus Acanthamoeba [1,2]. Yearly cases have continued to increase over time, with 43 cases per year in 2003, and 170 cases per year diagnosed in 2007 across thirteen ophthalmology centers in the USA [3]. AK remains a particularly challenging infection to diagnose and treat in a timely manner, due to its nonspecific clinical presentation and poor microbiologic isolation rate [1]. The ability of Acanthamoeba to present in two distinct forms—an active, infectious trophozoite form and a dormant, element-resistant cyst form—contributes to its infectivity and difficulty in eradication [2]. AK is frequently mistreated as a herpetic disease with concomitant use of steroids [1,2]. Delayed recognition and advanced disease progression often leads to significant visual loss and ocular morbidity [4,5]. The current gold standard for AK diagnosis is via a corneal culture. However, the sensitivity of this diagnostic modality is variable and limited, ranging from 33 to 67% [6,7,8]. In vivo confocal microscopy (IVCM) has emerged as an ancillary test to the corneal culture and has been known to improve diagnostic accuracy. However, limitations to its use include the learning curve involved, as well as its financial cost and availability [4].

Standard treatment of AK includes a biguanide, such as polyhexamethylene biguanide (PHMB) or chlorhexidine, with or without a diamidine such as propamidine isethionate or dibromopropamide [4]. Unfortunately, the treatment course for AK is often prolonged and challenging. Many patients require further intervention beyond medical treatment alone [4,8]. In a study on 227 patients, Papa et al. found only a 61% cure rate at 12 months for AK patients treated with standard medical therapy [9]. Alternative adjunct therapies such as Rose Bengal Photodynamic Antimicrobial Therapy (RB-PDAT) has emerged as a novel treatment for severe cases of infectious keratitis [10,11,12]. Importantly, RB-PDAT can be utilized in cases refractory to medical treatment or prior to surgical intervention. Naranjo et al. reported a 72% success rate of RB-PDAT amongst patients with infectious keratitis, of which 10/17 (59%) were infected with Acanthamoeba [10]. Specifically, 7/10 patients (70%) with AK resistant to medical therapy were treated successfully with RB-PDAT, while 3/10 patients (30%) underwent surgical intervention with a therapeutic penetrating keratoplasty (TPK). Similarly, a study by Sepulveda-Beltran et al. found a 78% success rate amongst 18 patients’ eyes with Acanthamoeba keratitis. Specifically, 14/18 (78%) did not require a penetrating keratoplasty, while 4/18 (22%) underwent surgical intervention with a TPK [11]. Despite a small sample size, the high success rate of patients with AK treated with RB-PDAT shows promise, given the known difficulty of eradication of the highly drug-resistant cystic form of AK. As early recognition of AK has been shown to be an important factor in the treatment of AK, perhaps there is a correlation between the penetrative capacity of the treatment modality within the cornea and depth of the resistant Acanthamoeba cyst.

The previous literature has suggested an association between Acanthamoeba cyst depth in the corneal layers and the severity of AK [2,13,14]. However, few studies have specifically quantified the Acanthamoeba cyst depth and cyst number in relation to patient prognosis [14]. In this study, we aimed to quantify these variables by analyzing human corneal tissue of patients with severe AK who underwent RB-PDAT and had a subsequent TPK, optical penetrating keratoplasty (OPK), or deep anterior lamellar keratectomy (DALK). Our objective was to gain insight into these factors to (1) allow for further understanding of the histopathologic findings in AK, (2) provide valuable clinical insights in prognostication, infection recurrence, and treatment resistance, and (3) shed light on the potential penetrative capacity of RB-PDAT in human corneas infected with *Acanthamoeba*.

## 2. Materials and Methods

### 2.1. Study Design and Setting

The study was a retrospective consecutive case series derived from the Bascom Palmer Eye Institute, Miami, FL, USA.

### 2.2. Study Population

Ethics committee approval was obtained from the Institutional Review Board of the University of Miami. All protocols adhered to the tenets of the Declaration of Helsinki. Medical records from a computerized database were reviewed between August 2016 to August 2022. All patients with a diagnosis of AK who underwent treatment via TPK, OPK, or DALK were included in this study. Each patient was deemed to be resistant to standard medical therapy and underwent subsequent treatment with RB-PDAT. Resistance to standard medical therapy was defined as clinical worsening or lack of improvement of clinical findings following standard medical therapy for a period of two weeks, as agreed upon by two ophthalmologists at Bascom Palmer Eye Institute. Written informed consent was obtained from all patients prior to RB-PDAT and surgical treatment. Demographic data collected included patient age, sex, past medical history, affected eye, and contact lens wear. A complete ophthalmic examination was performed at each follow-up visit. Outcome data included the organism cultured, type of surgical intervention performed, *Acanthamoeba* cyst depth, number of cysts, and mean corneal thickness.

### 2.3. Study Definitions and Outcome Measures

All patients included in this study were categorized as having severe AK, defined as patients who did not show any signs of clinical improvement after two weeks of standard medical therapy for AK. Primary outcome measures included assessment of factors such as Acanthamoeba cyst number and depth from Descemet’s layer, and corneal button thickness. Secondary outcome measures included mean follow-up time, presence of reinfection of the cornea, and subsequent type of reinfection, if reinfection occurred.

### 2.4. Rose Bengal Photodynamic Antimicrobial Therapy Procedure

RB-PDAT was performed according to previous studies by Naranjo et al., Sepulveda-Beltran et al., and Amescua et al. [10,11,15]. Briefly, a sterile lid speculum was placed under topical anesthesia (sterile lidocaine 1% and proparacaine 0.5%). If ulcers had a small epithelial defect, the area surrounding it was debrided to obtain an 8 mm de-epithelialized area to increase RB absorption. An 8 mm corneal sponge (Beaver Visitec International, Waltham, MA, USA) was placed over the cornea. Three drops of RB solution were applied to the sponge surface, followed by one drop every 3 min over the following 30 min to maintain saturation. Afterwards, the anterior corneal surface was irradiated with a custom-made 6 mW/cm^2^ green LED light source for 15 min for a total energy density exposure of 5.4 J/cm^2^. The anterior corneal surface was irrigated with one drop of balanced saline solution (Alcon Laboratories, Fort Worth, TX, USA) every 3 min throughout the light exposure to prevent corneal dehydration. Finally, a bandage contact lens (Airoptix AQUA; Alcon, Fort Worth, TX, USA) was placed to protect the ocular surface at the conclusion for pain management after the procedure and the ocular surface was examined at the slit lamp. Patients were evaluated after one, three, and seven days after procedure and reevaluated after two weeks of treatment to determine if they would benefit from another RB-PDAT session. If no significant signs of clinical improvement were seen, and physicians agreed that there was some benefit, an additional RB-PDAT treatment was given. Patients were afterwards followed biweekly, weekly, or monthly depending on their clinical progression. Patients continued their anti-amoebic medical therapy after RB-PDAT until the infection clinically resolved. In patients who still showed no clinical improvement, or in whom keratoplasty was considered the best next step, the decision to perform a TPK was made.

### 2.5. Histopathologic Analysis

The medical, histopathologic, and microbiologic records of all culture-proven cases of AK were analyzed following TPK, OPK, or DALK surgery. Histopathologic diagnosis of human corneal tissue infected with Acanthamoeba was confirmed by a board-certified pathologist (SRD) at the University of Miami, Bascom Palmer Eye Institute. Surgical specimens were stained with hematoxylin, eosin, and Periodic Acid-Schiff stains. Analysis of Acanthamoeba cyst depth, number of cysts, and average corneal thickness (anterior–posterior) was established by light microscopy. Cyst depth was determined by the difference between the distance of the most posterior cyst to Descemet’s membrane and the average total corneal thickness. Variable thickness of the corneal tissue limited anterior–posterior measurements of cyst depth, thus the distance from Descemet’s membrane was implemented.

### 2.6. Microbiologic Analysis

All patients with corneal ulcers underwent standard corneal scraping and microbiologic evaluation as per institutional protocol at the Bascom Palmer Eye Institute. Corneal scrapings and/or tissues were inoculated on to Non-Nutrient agar (NNA) overlaid with Escherichia coli, chocolate agar, 5% sheep blood agar, sabouraud agar, and thioglycollate agar. NNA and sabouraud were incubated at 35 °C ambient temperature and chocolate, blood, and thioglycollate in 35 °C in CO_2_ for up to 14 days. Gram and/or giemsa smears were examined for the presence of amoeba trophozoites and cysts. NNA plates were examined daily for the present of trophozoites (active “tracking”) or the presence of doubled wall cysts. Ninety-five percent were detected within 72 h and the remaining within seven days. For patients that underwent an OPK or DALK procedure, the removed corneal tissue did not undergo microbiological analysis at the time of surgery due to the lack of clinical signs of infection in these patients.

### 2.7. Statistical Analysis

The main outcome measure was the average Acanthamoeba cyst depth from Descemet’s layer and corneal button thickness per type of surgical intervention (TPK, or OPK + DALK). Statistical analyses were performed using SPSS version 29.0.2.0 (SPSS, IBM corporation, Armonk, NY, USA). An unpaired *t*-test was performed to compare number of Acanthamoeba cysts, average Acanthamoeba cyst depth from Descemet’s layer, and corneal button thickness per type of surgical intervention (TPK, or OPK + DALK). Due to a small sample size, patients undergoing an OPK or a DALK were combined for the purposes of statistical analysis. A *p*-value of <0.05 was considered statistically significant.

## 3. Results

### 3.1. Participant Characteristics

**Figure 1:**
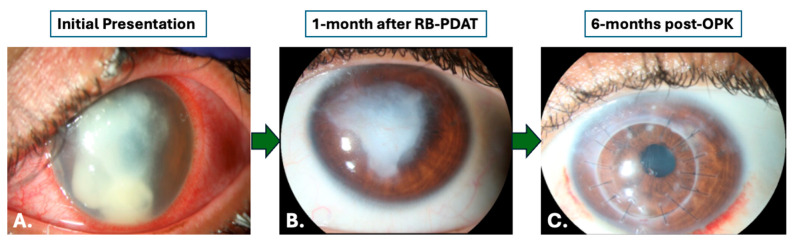
Slit lamp photos upon initial presentation, one month after treatment with Rose Bengal Photodynamic Antimicrobial Therapy, and six months after treatment with an optical penetrating keratoplasty for patients with *Acanthamoeba* keratitis. (**A**). Slit lamp photo upon initial presentation. (**B**). Slit lamp photo one month following RB-PDAT treatment. (**C**). Slit lamp photo six months after an optical penetrating keratoplasty.

**Figure 2:**
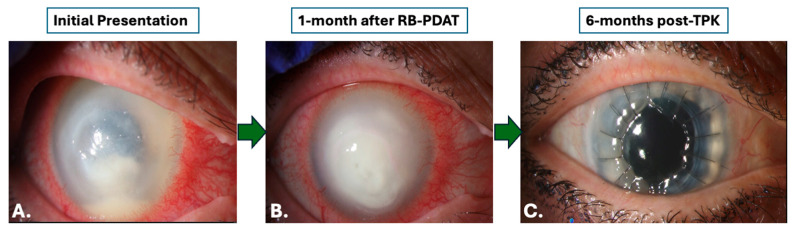
Slit lamp photos upon initial presentation, one month after treatment with Rose Bengal Photodynamic Antimicrobial Therapy, and six months after treatment with a therapeutic penetrating keratoplasty for patients with *Acanthamoeba* keratitis. (**A**). Slit lamp photo upon initial presentation. (**B**). Slit lamp photo one month following RB-PDAT treatment. (**C**). Slit lamp photo six months after a therapeutic penetrating keratoplasty.

A total of 17 patients underwent histopathologic and microbiologic analysis following treatment with RB-PDAT and TPK/OPK/DALK surgery for severe AK refractory to standard medical therapy at the Bascom Palmer Eye Institute between August 2016 and August 2022. Twelve patients (71%) were female and fifteen patients (88%) were contact lens users. The left eye was the affected eye in 10 patients (59%). Demographic and clinical characteristics are described in Table 1. A total of eight patients (47%) underwent TPK, seven patients (41%) underwent OPK, and two patients (12%) underwent DALK. Slit lamp images were collected at time of presentation, one month following RB-PDAT, and six months after surgical intervention (Figure 1 and Figure 2). Mean age was 44 ± 16 years old (range 17-to-65 years old) and overall mean time from symptom onset to the time of AK diagnosis was 59.6 ± 91.9 days. By type of procedure, mean time from symptoms onset to AK diagnosis was 78.1 ± 114.3 days and 35.0 ± 35.1 days for the OPK/DALK group and TPK group, respectively. Mean follow-up time was 30.3 ± 15.0 months.

### 3.2. Treatment

16 patients (94%) received treatment with chlorhexidine 0.02% ophthalmic solution. PHMB 0.02% ophthalmic solution was administered in 13 patients (77%). Specifically, seven patients (78%) received PHMB in the OPK/DALK group and six patients (75%) received PHMB in the TPK group. Miltefosine 50 mg caps were used in seven patients (41%) with an orphan drug designation for treatment of AK by the US Food and Drug Administration (FDA). Specifically, five (63%) of these patients underwent a subsequent TPK, and two (22%) patients underwent a subsequent OPK/DALK. The details of the treatments initiated are provided in Table 2.

### 3.3. Microbiologic and Histopathologic Outcomes

**Figure 3:**
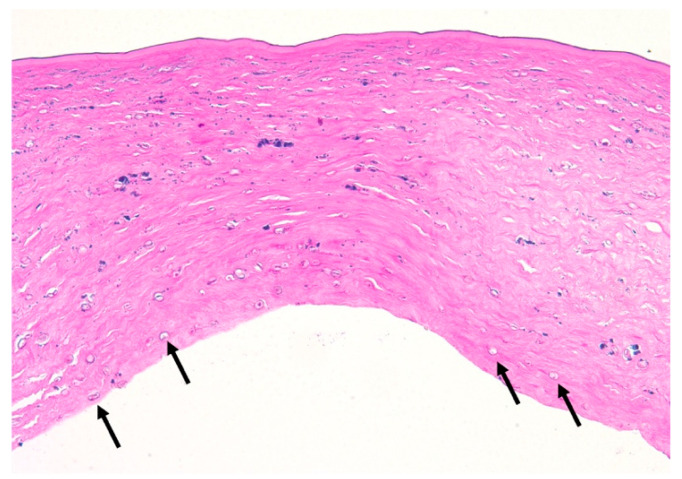
Hematoxylin, eosin, and periodic acid schiff (PAS) stained corneal tissue following therapeutic penetrating keratoplasty for progressive *Acanthamoeba* Keratitis. *Acanthamoeba* cysts (arrows) with variably Periodic Acid-Schiff positive cyst walls are present and extend up to full thickness with markedly edematous stroma. The epithelium and Descemet’s membrane are absent within this section (PAS, original magnification ×200).

**Figure 4:**
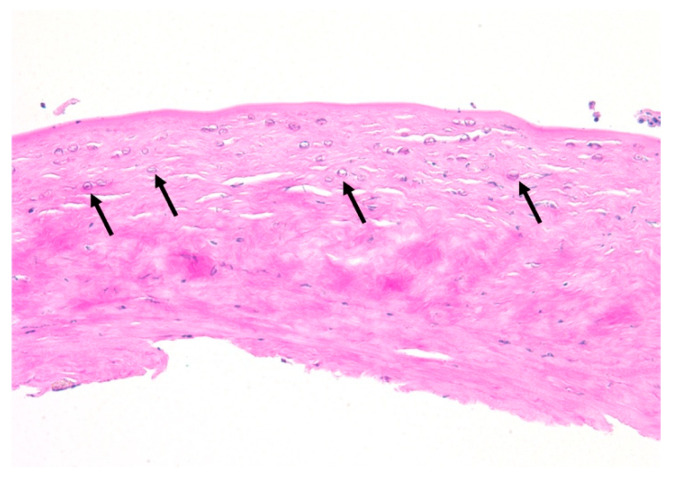
Hematoxylin, eosin, and Periodic Acid-Schiff (PAS) stained corneal tissue following optical penetrating keratoplasty for progressive *Acanthamoeba* keratitis. *Acanthamoeba* cysts (arrows) with variably Periodic Acid-Schiff positive cyst walls are present within markedly edematous and variably necrotic stroma and extend up to approximately 25% thickness. The epithelium and Descemet’s membrane are absent within this section (PAS, original magnification ×200).

A total of fourteen patients (82%) were diagnosed with AK via corneal scraping and three patients (18%) via corneal biopsy. Confocal microscopy was utilized to confirm the presence of *Acanthamoeba* in five out of eight TPK patients (63%). Of the five TPK patients that underwent confocal microscopy, three patients were positive for cysts, one patient was unclear/non-diagnostic, and one patient was negative for cysts (Table 3). Subsequent corneal tissue pathology analysis of the eight patients that underwent TPK revealed that six (75%) patients were positive for cysts (Figure 3). Amongst these patients, the average *Acanthamoeba* cyst depth from Descemet’s layer was 42.0 ± 52.5 μm (range 1–150 μm), the average corneal button thickness was 661.7 ± 106.5 μm, and the rate of reinfection after TPK was 13% (n = 1/8) (Table 3). Following TPK, the patient corneal host buttons were cultured for *Acanthamoeba*. A total of three (38%) patient cultures were positive for *Acanthamoeba* and five (63%) were negative. Specifically, three of the five patients (60%) that underwent adjuvant treatment with systemic miltefosine had negative cultures. Comparatively, of the nine patients that underwent OPK/DALK, only two underwent confocal microscopy. One patient was positive for cysts and one patient had an unclear/non-diagnostic confocal microscopy result (Table 3). Subsequent corneal tissue pathology analysis of the OPK/DALK group revealed three (33%) patients were positive for cysts on corneal tissue pathology analysis (Figure 4). Amongst the three patients, average *Acanthamoeba* cyst depth from Descemet’s layer was 261.7 ± 222.7 μm (range 25–560 μm). The average corneal button thickness was 474.2 ± 126.6 μm. Zero reinfections occurred after OPK/DALK. (Table 2). It was presumed that the AK was eradicated in patients undergoing optical correction via an OPK/DALK. Thus, most patients (n = 7; 78%) did not undergo corneal button culture analysis at the time of surgery. However, to verify our presumption, cultures were performed on two patients, both of which were negative for *Acanthamoeba* (Table 3). Significant differences were observed between TPK and OPK/DALK groups for (1) mean *Acanthamoeba* cyst depth from Descemet’s layer (*p* = 0.016), and (2) mean corneal button thickness *p* = 0.0051.

## 4. Discussion

The current study evaluates the microbiologic and histopathologic features of *Acanthamoeba* isolates recovered from patients with AK who underwent a TPK or OPK/DALK after treatment with RB-PDAT. In patients that underwent RB-PDAT followed by a TPK, *Acanthamoeba* cysts were found deeper within the cornea (mean depth from Descemet’s layer = 42.0 ± 52.5 μm) compared to patients that underwent RB-PDAT followed by an OPK/DALK (mean depth from Descemet’s layer = 261.7 ± 222.7 μm). Additionally, corneal button thickness was greater in patients that underwent TPK compared to patients undergoing an OPK/DALK (661.7 ± 106.5 μm vs. 474.2 ± 126.6 μm, respectively). We hypothesize that there may be a relationship between the depth of the *Acanthamoeba* cysts within the cornea and the severity of the infection, as suggested by a greater cyst depth in patients undergoing emergent, eye-saving TPK compared to cyst depth in patients undergoing an elective OPK/DALK for visual correction. Potential mechanisms of infection progression despite medical therapy may be due to difficulties in the penetrative capacity of standard medical therapy and RB-PDAT in patients with deep *Acanthamoeba* cysts.

Similar findings have been reported in the literature to date [13,14,16,17]. A study by Yokogawa et al. using IVCM on 18 eyes with AK found that most *Acanthamoeba* cysts were confined to the epithelial cell layer in patients with nonpersistent AK. However, in patients with persistent AK, cysts were found in both the epithelial layer and Bowman’s layer [13]. Additionally, Huang et al. characterized the density, depth, and distribution of *Acanthamoeba* cysts using IVCM on 21 eyes of 18 patients with AK. They reported an average cyst depth of 164.3 ± 81.2 μm overall and found a statistically significant difference in the cyst depth in patients that required TPK vs. standard medical therapy only (270.5 ± 17.5 μm vs. 139.4 ± 68.6 μm, respectively) [14]. They concluded that eyes infected with AK that required a subsequent TPK were more likely to have a deeper and more diffuse *Acanthamoeba* cyst penetration in the cornea compared to cases that resolved with standard medical therapy.

Our study corroborates previous findings and provides some insights into the penetrative capacity of RB-PDAT in human eyes with AK. In this study, RB-PDAT was deemed unsuccessful in patients that underwent emergent TPK. One possible hypothesis to explain this may be due to the inability of RB-PDAT to penetrate and target deeply located *Acanthamoeba* cysts, thus leading to infection progression and subsequent TPK. Conversely, amongst the OPK/DALK group, RB-PDAT showed success in targeting *Acanthamoeba* cysts located superficially within the cornea. Alternatively, another hypothesis may be that RB-PDAT combined with standard medical therapy was successful in eradicating the *Acanthamoeba* infection amongst the TPK group; however, the ophthalmologist’s decision to perform a TPK may have been due to the patient’s inflammatory response from treatment rather than an ongoing *Acanthamoeba* infection. This may be plausible given that only 38% (n = 3) of host corneal button cultures were positive for *Acanthamoeba* amongst patients undergoing a TPK. The previous literature has reported on increasing inflammation following the use of various treatment regimens for the treatment of AK [18,19]. A study by Thulasi et al. on 15 patients with AK treated with adjuvant systemic miltefosine treatment found that 73.3% of patients developed worsening inflammation after starting miltefosine. One patient underwent a penetrating keratoplasty due to significant thinning caused by inflammation from miltefosine [18]. Similarly, Naranjo et al. reported a variable inflammatory reaction observed amongst six patients with AK treated with systemic miltefosine [19]. We hypothesize that the inflammatory reaction following use of RB-PDAT and miltefosine may be due to increased immunological activity against antigenic material released during *Acanthamoeba* cell death [19].

To date, the exact depth of corneal penetration of RB-PDAT remains unknown and limited to non-inflamed and non-infected eyes of animal models [20,21,22,23,24]. In a study on RB-PDAT in rabbit eyes, Cherfan et al. found that 0.1% rose bengal applied to de-epithelialized corneas penetrated 100 μm into the corneal stroma [20]. Wertheimer et al. found that an Arginine-enhanced anaerobic crosslinking process increased penetration of RB into the stroma in rabbit eyes by 25% (~120 μm into the corneal stroma) compared to standard crosslinking with RB alone [21]. A study by Naranjo et al. on three rabbit eyes reported superficial RB-PDAT penetration. However, cellular apoptosis was evidenced in up to 1/3 of the stromal thickness, suggesting that oxidative stress produced via activation with green light can reach deeper into the corneal stroma [22]. Martinez et al. reported a mean corneal depth of 141 μm in rabbits treated with RB-PDAT visualized with anterior segment optical coherence tomography (AS-OCT) [23]. Lastly, a study by Aribas et al. on 45 rabbit eyes found that the application of iontophoresis-assisted RB-PDAT caused an increase in RB diffusion depth in the cornea to 42.22 ± 4.77% of the total corneal thickness compared to 26.63 ± 3.84% in RB-PDAT alone [24]. In our study, we hypothesize that RB-PDAT is capable of penetrating to the anterior to mid-stroma.

Limitations of this study include its small sample size and retrospective design. Further, not all patients were subject to the same medical treatment regimen. Despite these limitations, we believe the data presented will be useful for further understanding AK histopathology and RB-PDAT, as the performance of large sample studies remains challenging given the low incidence of AK across the USA. Overall, this study reports on *Acanthamoeba* cyst depth and quantity in patients with AK treated with RB-PDAT. This study details the histopathological findings in AK of varying severity and proposes a potential association between *Acanthamoeba* cyst depth and infection severity. These findings may provide valuable clinical insights in prognostication, as well as towards understanding infection recurrence and treatment resistance. Indirectly, this study sheds light on the potential penetrative capacity of RB-PDAT in human corneas. New technologies, such as iontophoresis-assisted RB-PDAT, show promise as potential options to improve the penetration of RB-PDAT.

## 5. Patents

University of Miami, HD, MCA, DM, GA, JMP have IP/patent related to the RB-PDAT technology.

## Figures and Tables

**Table 1:** Demographic data for patients who underwent surgical intervention for treatment-resistant *Acanthamoeba* keratitis.

Parameter	
**Gender, no. (%)**	
Male	5 (29)
Female	12 (71)
**Affected eye, no. (%)**	
Right	7 (41)
Left	10 (59)
**Age at time of surgical intervention (years)**	
Mean, (Standard Deviation)	44 (16)
Median	44
Range	17–65
**Type of surgical intervention, no. (%)**	
Therapeutic Penetrating Keratoplasty	8 (47)
Optical Penetrating Keratoplasty	9 (41)
Deep Anterior Lamellar Keratoplasty	2 (12)
**Follow-up time, months**	
Mean, (Standard Deviation)	30 (15)
**Contact lens user, no. (%)**	15 (88)

**Table 2:** Medical treatment received by patients with *Acanthamoeba* keratitis by type of surgical intervention.

Patient Group	Treatment, No. (%)			
	Chlorhexidine 0.02% Ophthalmic Solution	Polyhexamethylene Biguanide 0.02% Ophthalmic Solution	Voraconazole 50/200 mg Tablet	Miltefosine 50 mg Caps
**All patients (n = 17)**	16 (94)	13 (77)	7 (41)	7 (41)
**TPK only (n = 8)**	7 (88)	6 (75)	4 (50)	5 (63)
**OPK/DALK only (n = 9)**	9 (100)	7 (78)	3 (33)	2 (22)

**Table 3:** Microbiologic and histopathologic findings for patients who underwent surgical intervention for resistant *Acanthamoeba* keratitis.

Parameter	
**Method of Diagnosis of *Acanthamoeba* Keratitis, no. (%)**	
Corneal scrape	14 (82)
Corneal biopsy	3 (18)
**Therapeutic Penetrating Keratoplasty Group, no. (%)**	
Confocal microscopy findings:	
Positive for cysts	3 (38)
Unclear/non-diagnostic	1 (13)
Negative	1 (13)
Confocal not performed	3 (38)
Positive for cysts on corneal tissue pathology analysis	6 (75)
Culture of corneal host button at time of surgery:	
Positive for *Acanthamoeba*	3 (38)
Negative for *Acanthamoeba*	5 (63)
Culture not performed	0 (0)
Average *Acanthamoeba* cyst depth from Descemet’s layer	42.0 ± 52.5 μm
Average number of grading cysts	1.3 ± 0.7
Average corneal button thickness	661.7 ± 106.5 μm
Reinfection following surgical intervention	1 (13)
Time from symptom onset to AK diagnosis	35.0 ± 35.1 days
**Optical Penetrating Keratoplasty and Deep Anterior Lamellar Keratoplasty Group, no. (%)**	
Confocal microscopy findings:	
Positive for cysts	1 (11)
Unclear/non-diagnostic	1 (11)
Negative	0 (0)
Confocal not performed	7 (78)
Positive for cysts on corneal tissue pathology analysis	3 (33)
Culture of corneal host button at time of surgery:	
Positive for *Acanthamoeba*	0 (0)
Negative for *Acanthamoeba*	2 (22)
Culture not performed	7 (78)
Average *Acanthamoeba* cyst depth from Descemet’s layer	261.7 ± 222.7 μm
Average number of grading cysts	1.3 ± 0.9
Average corneal button thickness	474.2 ± 126.6 μm
Reinfection following surgical intervention	0 (0)
Time from symptom onset to AK diagnosis	78.1 ± 114.3 days
***p*-values following unpaired *t*-tests comparing TPK to OPK/DALK**	
Average *Acanthamoeba* cyst depth from Descemet’s layer	0.016
Average number of grading cysts	1.000
Average corneal button thickness	0.005

## Data Availability

The data that support the findings of this study are available from the corresponding author upon reasonable request.
